# Concurrent moxifloxacin-induced liver injury and toxic epidermal necrolysis after immune checkpoint inhibition: a case report and literature review

**DOI:** 10.3389/fimmu.2026.1753434

**Published:** 2026-03-25

**Authors:** Qiangsheng Li, Long Wang, Han Xu, Ting Huang, Haining Hong, Xiang Ji, Jun Liu

**Affiliations:** 1 Department of Thoracic Surgery, The Third People's Hospital of Bengbu Affiliated to Bengbu Medical University, Bengbu, Anhui, China; 2 Department of Pharmacy, The Third People's Hospital of Bengbu Affiliated to Bengbu Medical University, Bengbu, Anhui, China; 3 Department of Medical Oncology, The Third People's Hospital of Bengbu Affiliated to Bengbu Medical University, Bengbu, Anhui, China

**Keywords:** drug-induced liver injury, Stevens–Johnson syndrome, toxic epidermal necrolysis, immune checkpoint inhibitors, moxifloxacin

## Abstract

**Background:**

The widespread clinical use of immune checkpoint inhibitors (ICIs) in oncology has been accompanied by an increased incidence of immune-related adverse events (irAEs). When ICIs are combined with other medications, the risk of drug-induced liver injury (DILI) and Stevens–Johnson syndrome/toxic epidermal necrolysis (SJS/TEN) may be amplified. Moxifloxacin, a fluoroquinolone antibiotic known to cause hepatic injury and cutaneous adverse reactions, is an uncommon culprit in the setting of prior ICI therapy but warrants vigilance for its potential to precipitate acute DILI and TEN.

**Methods:**

We report a case of a 58-year-old man with lung adenocarcinoma who achieved a major pathological response after neoadjuvant tislelizumab combined with carboplatin and docetaxel. Postoperatively, he developed severe pneumonia and was treated with moxifloxacin and vancomycin. He subsequently developed severe DILI and TEN. Causality assessment implicated moxifloxacin as the principal offending agent, with a RUCAM score of 9 (probable) and an ALDEN score of 5 (probable). In addition, we conducted a systematic review of 28 published cases of concurrent DILI and SJS/TEN to characterize drug classes and clinical features.

**Results:**

Ten days after initiation of moxifloxacin, the patient developed synchronous DILI and TEN, suggesting that prior immunotherapy may have lowered the threshold for immune tolerance and amplified T cell–mediated, drug-induced immune responses. At 23 months postoperatively, no tumor recurrence was observed. Our systematic review identified a relatively high representation of fluoroquinolones among cases of concurrent DILI and SJS/TEN.

**Conclusion:**

Prior exposure to ICIs may enhance drug-triggered immune responses and thereby increase the risk of DILI and SJS/TEN. Clinicians should exercise caution when prescribing fluoroquinolones to patients who are receiving or have recently received immunotherapy and should intensify surveillance for drug-related adverse reactions. Future studies are needed to elucidate the immunologic synergism between ICIs and concomitant medications in order to optimize clinical management and risk prediction.

## Introduction

Drug-induced liver injury (DILI) is a relatively common adverse drug reaction, whereas Stevens–Johnson syndrome (SJS) and toxic epidermal necrolysis (TEN) are rare but life-threatening conditions; their concurrence substantially increases mortality risk. The expanding use of immune checkpoint inhibitors (ICIs) in cancer therapy has been associated with a higher incidence of immune-related adverse events (irAEs) (1). Concomitant administration of ICIs with other drugs may further increase the risk of both DILI and SJS/TEN, complicating causal attribution and differential diagnosis (2).

Fluoroquinolones (e.g., moxifloxacin) have been associated with DILI as well as SJS/TEN, and in the context of ICI therapy these agents may amplify immune-mediated toxicities (3, 4). In the era of rapidly expanding immune checkpoint inhibitor (ICI) use, delineating how prior PD-1 blockade may shape subsequent drug hypersensitivity has immediate clinical relevance. Although isolated reports suggest that ICIs can create an “immune-susceptible” background that predisposes patients to severe toxicity from later drugs (e.g., SJS/TEN with hepatotoxicity after sequential pembrolizumab and osimertinib), postoperative cases triggered by commonly used antibiotics have been scarcely characterized. In our systematic review (search through October 1, 2025), we identified 27 detailed case reports of concurrent DILI and SJS/TEN and further stratified them by the temporal sequence of hepatic and cutaneous involvement (type A *vs* type B), offering a pragmatic framework for phenotype-aware monitoring and risk stratification. Against this literature background, our case adds a perioperative, PD-1–exposed scenario in which moxifloxacin showed strong causality support (RUCAM 9; ALDEN 5) and highlights the need for heightened vigilance when prescribing high-risk agents (e.g., fluoroquinolones) to patients recently exposed to ICIs, particularly during systemic inflammation and polypharmacy.

## Case description

**Figure 1:**
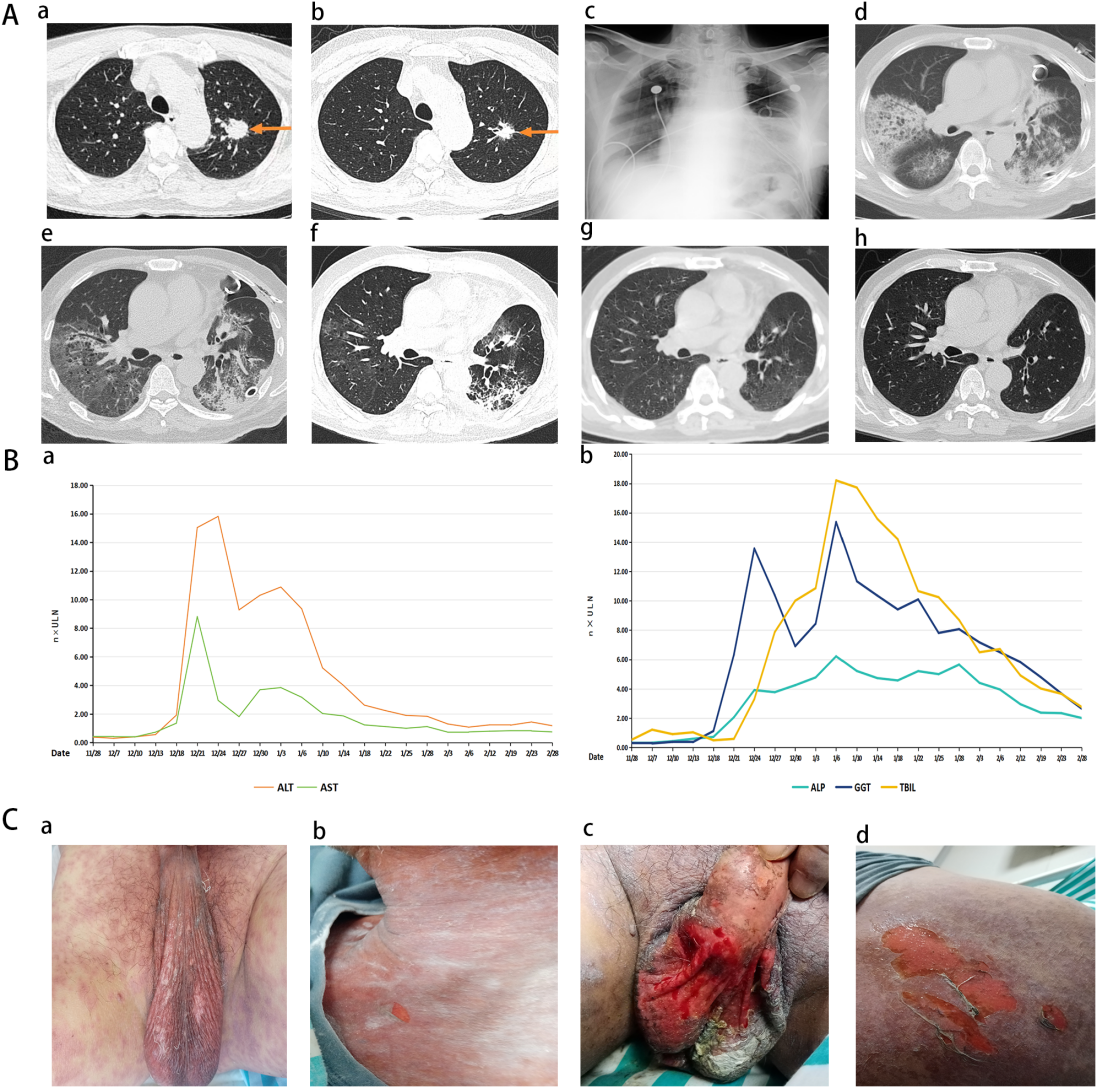
Temporal progression of key radiological, biochemical, and mucocutaneous findings during patient management. **(A)** Dynamic assessment of chest imaging. Sequential radiological images illustrate the progression of pulmonary lesions and inflammatory changes from preoperative neoadjuvant therapy to postoperative follow-up: **(a)** Two months prior to surgery (baseline): a left upper lobe nodule measuring 3.5 × 2.8 cm. The lung tumor lesion is indicated by the arrow; **(b)** One week before surgery (after two cycles of chemoimmunotherapy): marked reduction in the size of the pulmonary nodule; **(c)** Postoperative day (POD) 2: chest X-ray revealing left lower lobe inflammation with atelectasis; **(d)** POD 3: chest CT showing diffuse patchy infiltrates in both lower lobes; **(e)** POD 6: chest CT showing partial resolution of pulmonary inflammation; **(f)** POD 16: significant absorption of inflammatory infiltrates; **(g)** POD 30: complete resolution of pulmonary inflammation on radiological imaging; h POD 120: follow-up CT showing sustained remission with no recurrence. **(B)** Longitudinal changes in liver function parameters during treatment. The graph shows the longitudinal changes in alanine aminotransferase (ALT), aspartate aminotransferase (AST), alkaline phosphatase (ALP), γ-glutamyl transferase (GGT), and total bilirubin (TBIL). ALT peaked on POD 18 at 790 U/L (>15 × the upper limit of normal, ULN). ALP, GGT, and TBIL reached their respective peaks on POD 30 (ALP 783 U/L, 6.21 × ULN; GGT 1122 U/L, 15.37 × ULN; TBIL 400.6 µmol/L, 18.21 × ULN). **(C)** Clinical progression of severe cutaneous adverse reaction (TEN) at non-facial sites. **(a)** On POD 16, generalized erythematous rash with involvement of the perineal area; **(b)** On POD 20, epidermal detachment on the anterior chest; **(c–d)** On POD 22, further epidermal detachment and erosions in the perineal region, with the detachment extending over the posterior chest. In accordance with data protection policy, panels containing identifiable facial features (e.g., lips/oral region) have been removed. Oral mucosal erosions are described in the text but were not imaged for publication. Unfortunately, no photographs were taken during subsequent stages; however, the total affected body surface area exceeded 30%. TEN, toxic epidermal necrolysis; POD, postoperative day.

The patient was a 58-year-old man (BMI 26.3 kg/m²) with a 40 pack-year smoking history and a daily alcohol intake of approximately 200 g. His medical history included well-controlled type 2 diabetes and hypertension. He presented on September 8, 2023, with a 2-month history of cough and sputum production. Imaging revealed a mass in the left upper lobe of the lung (**Figure 1A-a**). CT-guided biopsy confirmed lung adenocarcinoma, clinically staged as cT2N2M0 (Stage IIIA).

**Figure 2:**
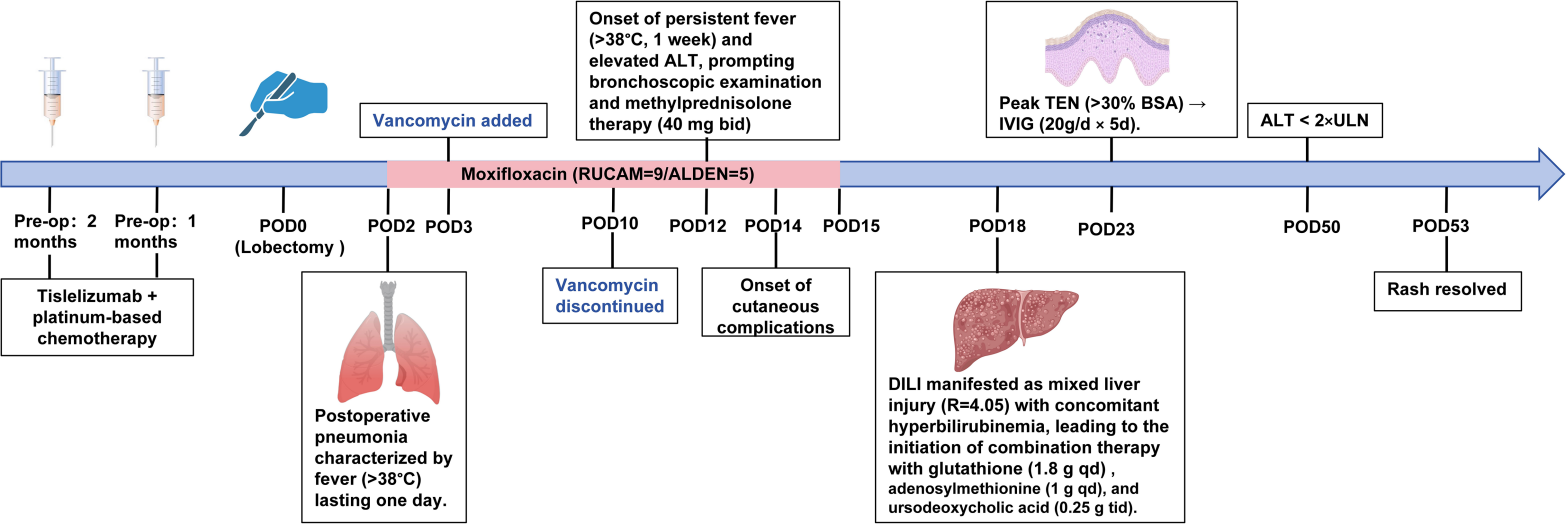
Key timeline events during treatment.

Key events in the clinical course, including the onset of complications and therapeutic interventions, are summarized in **Figure 2**. The patient received two cycles of neoadjuvant chemotherapy combined with immunotherapy (tislelizumab + carboplatin + docetaxel). The last tislelizumab dose (200 mg) was given on November 5, 2023, 31 days prior to lobectomy (December 6, 2023). Follow-up CT demonstrated a 68% reduction in tumor size compared with baseline and resolution of mediastinal lymph node metastasis (**Figure 1A-b**). He then underwent thoracoscopic left upper lobectomy with systematic lymph node dissection. Postoperative pathology revealed <10% residual viable tumor cells, consistent with a major pathological response (MPR).

On postoperative day (POD) 2, the patient developed high-grade fever (39.5°C) with leukocytosis. Bedside chest radiography showed left lower lobe infiltrates (**Figure 1A-c**), and severe pneumonia was diagnosed. Because of a documented β-lactam allergy, moxifloxacin was selected as the initial antibiotic therapy. On POD 3, chest CT revealed diffuse bilateral lower lobe infiltrates (**Figure 1A-d**). After consultation with the respiratory department, vancomycin was added to moxifloxacin, and methylprednisolone (1 mg/kg·d) was initiated. Vancomycin was discontinued on POD 10, and moxifloxacin was continued until POD 14. During this period, the patient’s temperature gradually normalized and imaging showed progressive improvement (**Figure 1A-e–f**).

On POD 12, during antimicrobial therapy, the patient developed abnormal liver function tests, with alanine aminotransferase (ALT) peaking at 790 U/L on POD 18. Total bilirubin further increased to 400.6 µmol/L on POD 31, indicating mixed-pattern DILI (**Figure 1B**; **Supplementary Table 1**). Serologic tests for viral hepatitis, autoimmune liver disease, and respiratory pathogens were negative.

Notably, on POD 14—two days after the onset of liver injury—the patient developed oral mucosal erosions, followed by a rapidly spreading erythematous rash and subsequent epidermal detachment, fulfilling the clinical criteria for TEN. In accordance with data protection policies, facial and oral photographs are not shown; the evolution of non-facial cutaneous lesions is presented in **Figure 1C-a–d**. Although photographs were not taken at later stages, the total body surface area affected by skin detachment was estimated to exceed 30%.

All nonessential medications were promptly discontinued. Hepatoprotective therapy with glutathione and adenosylmethionine was started, and methylprednisolone (1–2 mg/kg·d) plus antihistamines were continued. As skin lesions continued to progress by POD 23, intravenous immunoglobulin (IVIG, 20 g/d for 5 days) was added. Following this regimen, liver enzyme levels decreased by more than 50% within 2 weeks and returned to within twice the upper limit of normal within 4 weeks. The skin lesions entered the resolution phase within 10 days and fully re-epithelialized within 4 weeks. The patient was discharged in stable condition. At 23 months postoperatively, follow-up evaluations showed that he remained clinically stable with no evidence of tumor recurrence.

## Discussion

### Causality assessment and differential diagnosis

We applied the Roussel Uclaf Causality Assessment Method (RUCAM) to evaluate the causal relationship between each suspected drug and DILI. Moxifloxacin received a total RUCAM score of 9, corresponding to “highly probable,” indicating that the temporal relationship, improvement after drug withdrawal (dechallenge), and exclusion of alternative etiologies collectively support moxifloxacin as the most likely offending agent.

For TEN, we used the ALDEN algorithm (Algorithm of Drug Causality in Epidermal Necrolysis). Moxifloxacin received an ALDEN score of 5 (“probable”), further implicating it as the drug most strongly associated with TEN in this patient. Taken together with the clinical timeline, dechallenge response, and exclusion of alternative causes, these findings indicate that moxifloxacin best fulfills the criteria for the principal offending drug in this case (complete itemized scores and supporting evidence are provided in **Supplementary Tables 3, S4**).

By contrast, vancomycin, tislelizumab, docetaxel, and carboplatin each had RUCAM scores of 6, corresponding to a “possible” level of causality. Although vancomycin has been linked to hepatic injury in case reports, evidence for its role in causing severe multisystem toxicity remains limited (5, 6), and robust population-level data designating it as a high-risk DILI agent are lacking. Docetaxel and carboplatin have more frequently been associated with mild-to-moderate liver enzyme elevations, often in relation to tumor burden or pre-existing hepatic disease, without a characteristic drug-specific DILI phenotype.

Tislelizumab, a PD-1 inhibitor, carries a recognized risk of immune-related hepatitis (irH) (7). However, in our patient, tislelizumab was administered before moxifloxacin and no liver injury occurred during the initial immunotherapy course. On balance, we therefore consider tislelizumab more likely to have acted as a threshold-modulating or co-facilitating factor—lowering immune tolerance and reshaping T-cell responses—rather than as the direct trigger of the initial DILI and SJS/TEN events.

The patient reported chronic heavy alcohol intake (~200 g/day), which is recognized as a susceptibility factor in RUCAM and was incorporated into the risk-factor domain together with age ≥55 years (maximum contribution). However, despite the patient’s high alcohol intake, there was no clinical or laboratory evidence of chronic liver disease or baseline liver enzyme elevation prior to the toxic event (**Supplementary Table 1**). Importantly, we carefully excluded other potential causes of liver dysfunction — including viral hepatitis and autoimmune or other underlying liver diseases. In other words, alcohol likely served as a coexisting risk factor rather than an independent cause of the liver injury.

### Literature comparison and review

Using a predefined search strategy, we screened PubMed and Embase and identified 28 case reports of concurrent DILI and SJS/TEN (**Table 1**; Supplementary PRISMA diagram, **Supplementary Figure 1**). We conducted descriptive and comparative analyses to explore mortality, latency to onset, the relationship between prior drug-allergy history and SJS/TEN phenotype and mortality, and the characteristics and classification of implicated drugs, thereby providing preliminary insights into the clinical features and potential immunologic mechanisms underlying concurrent DILI and SJS/TEN.

**Table 1:** Reported cases of drug-induced liver injury (DILI) with concurrent SJS/TEN.

Case ID	Citation	Sex/Age	Allergy history	Culprit drug	Time to onset (days)	Onset sequence phenotype (A/B)	Liver injury pattern	Skin lesion	Outcome
1	Sheretz et al. (1985) (8)	49 y / M	None	Phenytoin	22	A	Hepatocellular	TEN	Survived
2	Chan et al. (1991) (9)	44 y / F	Allopurinol; Cefuroxime	Mefenamic acid	14	B (interval 14 days)	Cholestatic	SJS	Death (renal and hepatic failure)
3	Limauro et al. (1999) (10)	37y / M	None	Amoxicillin/clavulanic acid	32	A	Cholestatic	SJS	Death
4	Liu et al. (2001) (11)	62 y / F	Not reported	Ranitidine	9	A	Mixed	SJS	Recovered
5	Masia et al. (2002) (12)	47 y / M	Not reported	Clarithromycin + Disulfiram	7	A	Hepatocellular	TEN	Death
6	Nori et al. (2004) (13)	23 y / F	None	Moxifloxacin	3	A	Unknown	TEN	Death (despite LT)
7	Kakeda et al. (2005) (14)	68 y / M	Not reported	Allopurinol	23	B (interval 9 days)	Unknown	TEN	Death
8	Ohlmann et al. (2007) (15)	67 y / M	Not reported	Docetaxel	NA	B (interval 7 days)	Unknown	SJS	Death
9	Ekrief et al. (2007) (16)	37 y / F	Amoxicillin	Ibuprofen	3	B (interval 6 days)	Cholestatic	SJS	Recovered
10	Okan G et al. (2008) (17)	26 y / F	None	Ciprofloxacin	14	A	Mixed	SJS	Recovered
11	Domínguez-Leñero et al. (2009) (18)	66 y / F	None	Esomeprazole	11	B (interval 5 days)	Hepatocellular	SJS	Death
12	Jao et al. (2010) (19)	57 y / F	Not reported	Nevirapine	21	B (interval 5 days)	Hepatocellular	SJS	LT / survived
13	Yamaoka et al. (2012) (20)	76 y / F	Not reported	Etodolac	4	A	Unknown	TEN	Death
14	Patel et al. (2013) (21)	46 y / M	None	Nevirapine	56	A	Hepatocellular	SJS	Death
15	Budamakuntla et al. (2014) (22)	44 y / M	None	Nevirapine	20	B (interval 40 days)	Mixed	TEN	Recovered
16	Im et al. (2015) (23)	33 y / M	None	Lamotrigine	15	B (interval 3 days)	Hepatocellular	SJS	Death
17	Slim et al. (2015) (24)	29 y / F	None	Acetaminophen	3	B (interval 5 days)	Cholestatic	SJS	Recovered
18	Harimoto et al. (2015) (25)	40 y / F	Not reported	Acetaminophen	NA	B (interval unspecified)	Cholestatic	SJS	LT / survived
19	Agrawal et al. (2018) (26)	41 y / F	None	Fluoxetine	NA	A	Hepatocellular	SJS	Recovered
20	Shaikh et al. (2019) (27)	40 y / F	Not reported	oseltamivir	2	A	Mixed	TEN	Recovered
21	Totsuka et al. (2021) (28)	14 y / F	Not reported	Acetaminophen + Cefdinir	3	A	Hepatocellular	SJS	LT / survived
22	Gianni et al. (2021) (29)	54 y / F	Not reported	Osimertinib ( pembrolizumab exposure)	23	A	Unknown	SJS	Recovered
23	Gui et al. (2021) (30)	9 y / F	None	Ibuprofen	2	A	Unknown	SJS	Recovered
24	Xiong et al. (2021) (31)	61 y / M	Not reported	Warfarin	18	B (interval 7 days)	Unknown	SJS	Recovered
25	Lin et al. (2022) (32)	42 y / M	None	Diclofenac	3	B (interval 11 days)	Cholestatic	SJS	Death
26	Caviness et al. (2024) (33)	55 y / F	Not reported	Levetiracetam	21	A	Cholestatic	SJS	Recovered
27	Cheng et al. (2025) (34)	14 y / F	Not reported	Lamotrigine	15	A	Mixed	TEN	Chronic DILI
28	Our case (2025)	58 y / M	β-lactam allergy	Moxifloxacin (primary)	10	B (interval 2 days)	Mixed	TEN	Recovered

DILI, drug-induced liver injury; SJS, Stevens–Johnson syndrome; TEN, toxic epidermal necrolysis; LT, liver transplantation. Operational definition: Onset sequence phenotype: Phenotype A : simultaneous skin rash and liver injury. Phenotype B : skin rash before liver injury. Data harmonization: Liver injury patterns were classified as hepatocellular, cholestatic, or mixed when applicable. The outcomes were harmonized into categories of Survived, Liver transplant/survived, or Death. Time to onset was extracted as numeric days when explicitly reported; no imputation was performed. Deaths were classified as ‘Death’ or ‘Death (despite LT)’; ‘Liver transplant/survived’ was counted as survival; ‘Chronic DILI’ was considered non-fatal.

The overall case-fatality rate was 39.3% (11/28). Mortality was 36.8% in the SJS subgroup and higher in the TEN subgroup at 44.4% (4/9), consistent with the higher lethality of TEN reported in previous studies (35). However, given the small sample size, this difference was not statistically significant (relative risk ≈ 1.21; 95% CI, 0.47–3.08; Fisher’s exact p = 1.000).

The median latency to onset was 9 days (interquartile range [IQR] 3–14.5 days) for the overall cohort, 14 days (IQR 3–21) for SJS, and 10 days (IQR 4–20) for TEN. The somewhat shorter latency in the TEN subgroup suggests a potentially more acute clinical course in some patients, but this trend was not statistically significant (p = 0.772) and requires confirmation in larger cohorts.

We further reclassified the 28 cases according to the temporal sequence of cutaneous and hepatic involvement: type A (simultaneous onset of skin and liver involvement; n = 15) and type B (cutaneous manifestations preceding liver injury; n = 13). In type B cases, the interval between rash onset and liver injury ranged from 2 to 40 days, with a median of approximately 6–7 days; most patients developed liver injury within two weeks. Mortality was 33.3% (5/15) in type A and 46.2% (6/13) in type B (Fisher’s exact p ≈ 0.70). SJS predominated in both groups (type A: 60.0% SJS, 40.0% TEN; type B: 76.9% SJS, 23.1% TEN; Fisher’s exact p ≈ 0.43). Temporal subtyping thus offers a useful descriptive framework for cutaneous–hepatic involvement but, given the limited sample size, does not support firm conclusions regarding prognosis or rash phenotype.

Drug-distribution analysis showed a substantial representation of anti-infective agents—particularly fluoroquinolones—which accounted for 36.4% of implicated drugs; moxifloxacin specifically accounted for 13.6%. Other frequently implicated classes included nonsteroidal anti-inflammatory drugs (NSAIDs; 22.7%), antineoplastic agents (13.6%), and antiepileptics (9.1%). This distribution aligns with prior literature on drug-related SJS/TEN (6, 36), underscoring the prominent role of fluoroquinolones and other classical high-risk agents in cases of concurrent DILI and SJS/TEN. However, as most available evidence is derived from individual case reports and small series, precise comparative estimates of relative risk across drugs cannot be made. Larger, prospective pharmacovigilance studies are needed to validate these findings.

Only three patients had a clearly documented history of drug allergy, all to β-lactam antibiotics. This raises a clinically relevant question: in the context of polypharmacy, might a history of β-lactam allergy predispose patients to quinolone-induced DILI and/or SJS/TEN (e.g., from moxifloxacin)? Gelincik et al. (2005) reported that, although quinolones generally cause fewer hypersensitivity reactions than β-lactams, clear associations have been observed in a subset of patients (37). Doña et al. (2017) further demonstrated an increased risk of immediate-type hypersensitivity to quinolones among individuals with drug-provocation–confirmed β-lactam allergy (38). These studies, however, focus on IgE-mediated immediate hypersensitivity. Prospective or large-scale data are lacking regarding β-lactam allergy as an independent risk factor for quinolone- or NSAID-associated DILI and SJS/TEN. At present, we therefore interpret prior β-lactam allergy as a potential marker of immunologic susceptibility rather than a proven independent risk factor; its predictive value requires validation in large cohorts that account for multidrug exposures and the context of immunotherapy.

### Mechanistic considerations

A fundamental concept underpinning drug-specific T-cell responses is the pharmacological interaction (p-i) model, which posits that certain small-molecule drugs can noncovalently interact with T-cell receptors (TCRs) and human leukocyte antigen (HLA) molecules, thereby activating antigen-specific T cells (39). In the present case, the patient received the PD-1 inhibitor tislelizumab, subsequently developed severe postoperative pneumonia, and was treated with moxifloxacin, after which he developed DILI and TEN. The temporal pattern and clinical features are consistent with a T cell–mediated, drug-specific hypersensitivity mechanism. Fluoroquinolones, including moxifloxacin, have been shown to activate drug-specific T cells and exhibit cross-reactivity, lending biological plausibility to the notion that a single agent can elicit distinct organ-specific phenotypes (40, 41). Several non-mutually exclusive mechanisms may have contributed to disease pathogenesis in this patient.

### PD-1 inhibition and lowering of the immune-tolerance threshold

The PD-1 pathway is central to the maintenance of T-cell tolerance, the regulation of effector T-cell responses, and the function of regulatory T cells (42). PD-1 blockade lowers the activation threshold of T cells and attenuates inhibitory signaling, thereby potentiating immune responses. This modulation is more likely to affect the velocity and magnitude of drug-specific responses than to alter the fundamental mechanism (43, 44). Immune checkpoint inhibitor–related hepatic injury is characterized by prominent T-cell infiltration and macrophage activation, providing a biologic basis for amplified toxicity when a putative offending drug such as moxifloxacin is introduced into an immune-activated milieu (45). Although direct pharmacokinetic–pharmacodynamic data are lacking, PD-1 receptor blockade may exert functional effects that persist for weeks or longer after drug discontinuation, creating a relatively durable “window of immune hypersensitivity.”

### Postoperative pneumonia and systemic inflammation

Surgery and severe postoperative pneumonia are classic triggers of systemic inflammation and can disrupt immune homeostasis, promoting a hyperresponsive immune state (46). Under such conditions, T cells are more readily activated and drug–antigen interactions may more easily precipitate aberrant immune responses. Inflammatory mediators released during infection and surgical stress (e.g., IL-6, IL-10) can remodel hepatic and cutaneous immune microenvironments, thereby amplifying drug toxicity. In our patient, sustained immune activation due to postoperative pneumonia—superimposed on prior PD-1 blockade—may have substantially increased the risk of moxifloxacin-induced DILI and TEN. This mechanistic inference is based primarily on indirect evidence from inflammatory markers and requires validation in prospective immunologic studies.

### Potential impact of HPA-axis activation

Although the last dose of ICI was administered more than one month before surgery, the patient showed persistently elevated ACTH and cortisol levels preoperatively, suggesting sustained activation of the hypothalamic–pituitary–adrenal (HPA) axis. HPA-axis activation triggers cortisol release and modulates immune and inflammatory responses. However, during prolonged or intense stress, glucocorticoid resistance and impaired feedback may develop, blunting immunosuppressive effects and destabilizing immune regulation. In this case, persistent HPA-axis activation, together with PD-1–mediated lowering of immune tolerance and postoperative inflammation, may have created a “second-hit” state that further reduced the threshold for immune activation and facilitated moxifloxacin-induced T-cell cytotoxicity. Perioperative elevations in C4, IL-6, and IL-10 (**Supplementary Table 2**) mirror the acute pro-inflammatory/compensatory pattern and complement involvement described in SJS/TEN, providing supportive but non-specific evidence for this threshold-lowering hypothesis. In our case, direct evidence of glucocorticoid receptor function or downstream T-cell responsiveness is lacking, so this interpretation remains speculative. We therefore present the HPA axis activation and possible glucocorticoid resistance as a tentative hypothesis.

### Comparison with previously reported cases

Comparison with earlier reports offers additional mechanistic insight. Nori et al. (2004) described rapid progression to acute liver failure with concurrent TEN within approximately 3 days of moxifloxacin exposure in a patient who had not received ICIs, suggesting pre-existing sensitization and rapid reactivation of memory drug-specific T cells. In contrast, Gianni et al. (2021) reported an EGFR-mutant lung cancer patient who developed SJS/TEN and hepatic injury after sequential PD-1 blockade and subsequent osimertinib therapy, supporting the concept that ICIs can create an “immune-susceptible” or low-threshold background predisposing to severe toxicity from later drugs. Our case—occurring after PD-1 inhibition and in the setting of postoperative pneumonia and HPA-axis activation—had a latency of approximately 10 days, consistent with a primary drug-specific hypersensitivity response but within a complex perioperative immune environment.

Taken together, the Nori case may represent a rapid, memory T-cell–driven model, whereas ICI-associated cases (including that reported by Gianni et al. and ours) are more consistent with a dual-axis model involving a pharmacologic trigger plus immune-threshold modulation by ICI exposure and physiologic stressors. This framework may help explain the heterogeneous kinetic phenotypes of moxifloxacin-associated DILI plus SJS/TEN and suggests the potential value of time- and host-based risk stratification in ICI-treated populations.

### Review of high-risk factors

Previous studies have shown that the development of DILI depends not only on the offending drug but also on host-related risk factors, including advanced age, female sex, pre-existing or concomitant liver disease, metabolic syndrome and diabetes, heavy alcohol use, polypharmacy, and genetic susceptibility mediated by specific HLA alleles (47). These factors can substantially increase the risk of DILI when patients are exposed to hepatotoxic agents.

For SJS/TEN, major risk factors include genetic susceptibility (e.g., HLA-B15:02, HLA-B58:01), HIV infection, malignancy or autoimmune disease, chronic renal impairment, older age, and exposure to high-risk medications such as antiepileptics, allopurinol, certain antibiotics, and NSAIDs (48). Against this background, our patient had malignancy, prior ICI exposure, severe postoperative pneumonia, and concomitant use of several drugs with potential hepatic and/or cutaneous toxicity—constituting a typical “susceptible host plus high-risk drug exposure” profile and placing him at dual high risk for both DILI and SJS/TEN.

β-lactam antibiotics are among the most common culprit drugs for severe cutaneous adverse reactions (SCARs) (49). However, no prospective or large-scale studies have yet evaluated a history of β-lactam allergy as an independent risk factor in models of quinolone- or NSAID-associated DILI and SJS/TEN. In this case, such a history is more plausibly interpreted as a marker of underlying drug hypersusceptibility, while the principal triggers and amplifiers are more likely attributable to the combined effects of ICI-related lowering of the immune-tolerance threshold, postoperative inflammation, and moxifloxacin exposure.

## Clinical implications

This study provides an initial clinical characterization of concurrent DILI and SJS/TEN and proposes a hypothetical mechanism by which moxifloxacin may trigger mixed hepatic and cutaneous hypersensitivity reactions in the setting of prior PD-1 exposure. Our data support a dual-axis model in which a pharmacologic trigger interacts with modulation of the immune-tolerance threshold, suggesting that ICI exposure may lower immune tolerance and thereby amplify subsequent drug-induced, T cell–mediated immune responses. A limitation of this report is that histopathologic confirmation was not available, as liver and skin biopsies were not performed because informed consent/authorization for invasive sampling could not be obtained during the acute, severe phase of illness.

Although the number of available cases is small and some data are incomplete, our findings highlight the potentially high-risk role of fluoroquinolones in DILI accompanied by SJS/TEN and provide practical clues for clinical management. Clinicians should be cautious when prescribing drugs with known hepatic or cutaneous toxicity—particularly fluoroquinolones—to patients receiving or having recently received ICIs, especially in the presence of systemic inflammation or polypharmacy. Early recognition, prompt discontinuation of suspected agents, and timely initiation of supportive and immunomodulatory treatment are critical for improving outcomes.

Future research should focus on clarifying the synergistic mechanisms between ICIs and concomitant medications and on dissecting immunologic pathways such as drug-specific T-cell responses and HLA susceptibility alleles. Such studies may ultimately facilitate personalized risk stratification and more precise guidance for preventing and managing drug hypersensitivity and immune-mediated toxicities in cancer patients undergoing immunotherapy.

## Conclusion

We report a case of mixed-pattern drug-induced liver injury (DILI) and toxic epidermal necrolysis (TEN) attributed to moxifloxacin in the context of prior ICI therapy. Our observations suggest that prior ICI exposure may lower the threshold for immune tolerance, thereby amplifying drug-induced immune responses. We also highlight the potential contributions of postoperative systemic inflammation and HPA-axis activation to the development of immune-mediated toxicity.

These findings underscore the need for heightened vigilance when prescribing drugs with potential hepatic or cutaneous toxicity to patients who are receiving or have recently received immunotherapy, particularly in settings of immune activation and polypharmacy. Future studies should aim to delineate the immune synergism between ICIs and concomitant medications in order to refine clinical drug management, improve early risk identification, and prevent severe adverse reactions.

## Data Availability

The original contributions presented in the study are included in the article/**Supplementary Material**. Further inquiries can be directed to the corresponding author.
