# Supplementary material for: Concurrent moxifloxacin-induced liver injury and toxic epidermal necrolysis after immune checkpoint inhibition: a case report and literature review
Source: Front Immunol. 2026 Mar 25;17:1753434. doi: 10.3389/fimmu.2026.1753434 (PMC13057447; doi:10.3389/fimmu.2026.1753434)

| **Table S1 (Supplementary). Liver function changes during the treatment course** | | | | | |
| --- | --- | --- | --- | --- | --- |
| Date | ALT(≤50U/L) | AST(≤59U/L) | ALP(≤126U/L) | GGT(≤73U/L) | TBIL(≤22.0 µmol/L) |
| 2023/10/8 | 31 | 43 | 33 | 23 | 18.3 |
| 2023/11/4 | 24 | 35 | 76 | 33 | 10.3 |
| 2023/11/28 | 19 | 25 | 40 | 22 | 11.6 |
| 2023/12/06 (Lobectomy ) |  |  |  |  |  |
| 2023/12/7 | 15 | 24 | 41 | 20 | 26.7 |
| 2023/12/10 | 20 | 23 | 54 | 28 | 20 |
| 2023/12/13 | 28 | 43 | 75 | 27 | 22.8 |
| 2023/12/18 | 96 | 80 | 91 | 82 | 10.8 |
| 2023/12/21 | 752 | 520 | 259 | 461 | 12.9 |
| 2023/12/24 | 791 | 174 | 493 | 990 | 72.4 |
| 2023/12/27 | 464 | 107 | 475 | 758 | 173.1 |
| 2023/12/30 | 515 | 218 | 535 | 504 | 220.1 |
| 2024/1/3 | 544 | 227 | 602 | 616 | 238.9 |
| 2024/1/6 | 468 | 187 | 783 | 1122 | 400.6 |
| 2024/1/10 | 261 | 120 | 657 | 827 | 389.8 |
| 2024/1/14 | 200 | 110 | 596 | 756 | 342.9 |
| 2024/1/18 | 131 | 73 | 576 | 687 | 312.4 |
| 2024/1/22 | 112 | 66 | 656 | 737 | 234.6 |
| 2024/1/25 | 95 | 59 | 630 | 570 | 225.4 |
| 2024/1/28 | 92 | 66 | 712 | 589 | 191.3 |
| 2024/2/3 | 65 | 43 | 555 | 523 | 142.8 |
| 2024/2/6 | 54 | 44 | 498 | 474 | 147.6 |
| 2024/2/12 | 62 | 47 | 372 | 425 | 108.1 |
| 2024/2/19 | 61 | 49 | 299 | 350 | 88.4 |
| 2024/2/23 | 72 | 48 | 295 | 269 | 80.6 |
| 2024/2/28 | 59 | 44 | 253 | 193 | 60.7 |

“Baseline liver tests before chemo-immunotherapy (Oct 8 and Nov 4, 2023) and preoperative admission (Nov 28, 2023) were within normal ranges.”

| **Table S2. Perioperative Immune Milieu Over Time** | | | | |
| --- | --- | --- | --- | --- |
| Date (POD) | Analyte | Result | Reference range | Flag |
| 2023-11-04(Pre-first ICI) | ACTH | 88.4 pg/mL | 7.2–63.3 pg/mL | ↑ |
| 2023-11-04 (Pre-first ICI) | Cortisol (AM) | 640 nmol/L | 171–536 nmol/L | ↑ |
| 2023-11-28(Pre-second ICI) | Cortisol (AM) | 446 nmol/L | 171–536 nmol/L | mild ↑ |
| 2023-12-06 (Lobectomy ) |  |  |  |  |
| 2023-12-21 (POD15) | C3 | 1.58 g/L | 0.90–1.80 g/L | — |
| 2023-12-21 (POD15) | C4 | 0.636 g/L | 0.10–0.40 g/L | ↑ |
| 2023-12-28 (POD22) | IL-6 | 14.54 pg/mL | 0–5.30 pg/mL | ↑ |
| 2023-12-28 (POD22) | IL-10 | 6.19 pg/mL | 0–4.91 pg/mL | ↑ |
| ACTH: Adrenocorticotropic hormone；IL-6: Interleukin 6；IL-10: Interleukin 10；Flag: A symbol indicating abnormal results, with "↑" signifying an increase above the normal range and "mild ↑" indicating a moderate increase. | | | | |

| **Table S3. RUCAM Causality Assessment for Suspected Drugs in a Case of Mixed Liver Injury** | | | | | | | | | |
| --- | --- | --- | --- | --- | --- | --- | --- | --- | --- |
| **Suspected Drug** | **(I) Time to Onset** | **(II) Course** | **(III) Risk Factors** | **(IV) Concomitant Drugs** | **(V) Exclusion of Other Causes** | **(VI) Previous Hepatotoxicity** | **(VII) Rechallenge** | **Score (I-VII total)** | **Causality** |
| Moxifloxacin | +2 a | +2 b | +2 c | -1 d | +2 e | +2 f | 0 g | 9 | **Highly probable** |
| Vancomycin | +2 h | 0 k | +2 c | -1 d | +2 e | +1 i | 0 g | 6 | **Probable** |
| Tislelizumab | +2 j | 0 k | +2 c | -2 l | +2 e | +2 f | 0 g | 6 | **Probable** |
| Docetaxel | +2 j | 0 k | +2 c | -2 l | +2 e | +2 f | 0 g | 6 | **Probable** |
| Carboplatin | +2 j | 0 k | +2 c | -2 l | +2 e | +2 f | 0 g | 6 | **Probable** |
| Rocuronium | +2 m | 0 k | +2 c | -3 n | +2 e | 0 o | 0 g | 3 | Possible |
| Flumazenil | +2 m | 0 k | +2 c | -3 n | +2 e | 0 o | 0 g | 3 | Possible |
| Pentazocine | +2 m | 0 k | +2 c | -3 n | +2 e | 0 o | 0 g | 3 | Possible |
| Azasetron | +2 m | 0 k | +2 c | -3 n | +2 e | 0 o | 0 g | 3 | Possible |
| **Table footnotes:**  **a:** Latency from initiation of moxifloxacin to the index day of liver injury was 10 days (8 December to 18 December 2023), which lies within the typical 5–90-day window for drug-induced liver injury (DILI) in the RUCAM scale. **b**: Dechallenge for moxifloxacin: After withdrawal of moxifloxacin, ALP decreased by more than 50% within 180 days (from a peak of 783 U/L on 6 January 2024 to below half of this value by mid-February 2024), supporting a positive course and yielding a score of +2 for criterion II in the cholestatic/mixed RUCAM. **c:** Presence of risk factors: the patient was 58 years old (≥55 years) and had chronic excessive alcohol consumption (~200 g/day). According to RUCAM, the combination of advanced age and alcohol use confers the maximum risk factor score of +2. **d:** Mild impact of concomitant drugs (−1) for moxifloxacin and vancomycin: these agents were co-administered with a limited number of other potentially hepatotoxic drugs, but none provided a more convincing explanation for the liver injury than the assessed drug itself. Therefore, only a minor negative weight (−1) was applied for criterion IV. **e:** All non-drug causes of liver injury were reasonably ruled out, including viral hepatitis (hepatitis A, B, C, E), autoimmune liver diseases, biliary obstruction, and ischemic hepatitis. Laboratory tests (viral serologies, autoimmune panels) and imaging studies did not suggest alternative etiologies. **f:** Hepatotoxicity is well recognized for this drug or its class, either explicitly mentioned in the official product label and/or supported by multiple published case series. This applies to moxifloxacin, tislelizumab, docetaxel, and carboplatin, which all receive +2 for criterion VI. **g:** Rechallenge was not performed for any of the suspected drugs; therefore, criterion VII was scored as 0 in all cases. **h:** Latency from initiation of vancomycin to the index day was approximately 11–12 days, which lies within the 5–90-day window used by RUCAM for cholestatic/mixed injury; thus, criterion I for vancomycin scored +2. **i:** For vancomycin, hepatotoxicity has been reported in the literature (e.g., in the context of DRESS or multiorgan injury), but it is less frequently described than for the drugs scored with footnote f. Accordingly, criterion VI was assigned a score of +1. **j:** For tislelizumab, docetaxel, and carboplatin, the interval between the last neoadjuvant cycle (5 November 2023) and the index day of liver injury (18 December 2023) was about 6 weeks, which falls within the 5–90-day latency window specified by RUCAM; therefore, criterion I scored +2 for these agents. **k:** For vancomycin, tislelizumab, docetaxel, carboplatin, and perioperative drugs, the course (criterion II) could not be clearly ascribed to the individual drug, because several agents had been discontinued before the peak of liver injury, and the patient remained exposed to other suspected drugs during the worsening and recovery phases. Therefore, criterion II was conservatively scored as 0 in these cases. **l:** Moderate impact of concomitant drugs (−2) for tislelizumab, docetaxel, and carboplatin: these drugs were given in combination and later overlapped in time with moxifloxacin and vancomycin, both of which are strong alternative explanations for the observed liver injury and TEN. Consequently, a −2 penalty was applied to criterion IV for these neoadjuvant agents. **m:** Perioperative drugs (rocuronium, flumazenil, pentazocine, and azasetron) were administered as single or very short-term doses on the day of surgery (6 December 2023). The index day of liver injury was 18 December 2023, resulting in a latency of 12 days, which still falls within the 5–90-day range and yields +2 for criterion I. **n:** For rocuronium, flumazenil, pentazocine, and azasetron, the liver injury occurred in a setting of polypharmacy and exposure to stronger suspect drugs (especially moxifloxacin), while these agents were given only perioperatively as single doses. Under RUCAM, this warrants a marked negative impact (−3) for concomitant drugs in criterion IV. **o:** For the perioperative drugs, no convincing signal of clinically significant hepatotoxicity was identified in the product labels or in the available literature; thus, criterion VI was scored as 0. RUCAM Score Interpretation: ≥9: Highly probable; 6-8: Probable; 3-5: Possible; 1-2: Unlikely; ≤0: Excluded. Key to Assessment:The R-value was calculated using the peak values of ALT and ALP on 24 December 2023 (ALT 791 U/L, ALP 493 U/L), yielding an R-value of approximately 4.05, which is consistent with a mixed liver injury pattern. The index day of liver injury was defined as 18 December 2023, when liver tests first showed clear abnormalities and clinical symptoms of liver injury emerged. Because the injury pattern was mixed, the cholestatic/mixed RUCAM was applied, and the course of ALP (alkaline phosphatase) was used for criterion II. | | | | | | | | | |

| **Table S4. ALDEN Causality Assessment for Suspected Drugs in a Case of Stevens-Johnson Syndrome** | | | | | | | | |
| --- | --- | --- | --- | --- | --- | --- | --- | --- |
| **Suspected Drug** | **Administration Period** | **(I) Latency** | **(II) Drug Presence on Index Day** | **(III) Previous Exposure/Reaction** | **(IV) Drug Use After SJS Onset** | **(V) Notoriety** | **Score (I-V total → Final) ^a^** | **Causality** |
| Moxifloxacin | Dec 08 – Dec 21, 2023 | +3 b | 0 c | 0 d | 0 e | +2 f | 5 | **Probable** |
| Vancomycin | Dec 09 – Dec 15, 2023 | +3 b | -3 g | 0 d | 0 h | +1 i | 1 → 0 | Unlikely |
| Tislelizumab | Last dose: Nov 05, 2023 | -1 j | 0 k | 0 d | 0 h | +1 i | 0 → -1 | Excluded |
| Rocuronium | Single dose: Dec 06, 2023 | +3 b | -3 g | 0 d | 0 h | 0 l | 0 → -1 | Excluded |
| Flumazenil | Single dose: Dec 06, 2023 | +3 b | -3 g | 0 d | 0 h | 0 l | 0 → -1 | Excluded |
| Pentazocine | Single dose: Dec 06, 2023 | +3 b | -3 g | 0 d | 0 h | 0 l | 0 → -1 | Excluded |
| Azasetron | Single dose: Dec 06, 2023 | +3 b | -3 g | 0 d | 0 h | 0 l | 0 → -1 | Excluded |
| Docetaxel | Last dose: Nov 05, 2023 | -1 j | -3 g | 0 d | 0 h | 0 l | -4→ -5 | Excluded |
| Carboplatin | Last dose: Nov 05, 2023 | -1 j | -3 g | 0 d | 0 h | 0 l | -4→ -5 | Excluded |
| **Table footnotes:** **a** Score deduction for polypharmacy (Criterion VI) was applied uniformly (-1 point) to all drugs. The score is presented as (Subtotal I-V) → (Final Score after deduction)；**b** Latency between 5 and 56 days；**c** Drug was continued on the index day；**d** No previous history of exposure or reaction to the drug；**e** Drug was discontinued on the day of SJS onset (Dec 21, 2023)；**f** Drug is listed as suspected for SJS；**g** Drug was discontinued >5 half-lives before the index day；**h** Drug was discontinued before SJS onset；**i** A score of +1 was assigned for tislelizumab and vancomycin notoriety because SJS/TEN cases have been reported for this drug；**j** Latency >56 days；**k** Drug was discontinued <5 half-lives before the index day；**l** No strong notoriety for causing SJS/TEN. **ALDEN Score Interpretation**: ≥6: Very probable; 4-5: Probable; 2-3: Possible; 0-1: Unlikely; ≤0: Excluded. **Key Clinical Context**: Index Day (first day of rash): December 20, 2023. | | | | | | | | |

**Supplementary Figure S1. PRISMA 2020 flow diagram.** Searches of Embase and PubMed from inception to October 1, 2025 (case reports; keywords: “drug-induced liver injury”/“liver toxicity”/“liver failure” AND “Stevens–Johnson syndrome”/“toxic epidermal necrolysis”/“Stevens–Johnson/toxic epidermal necrolysis”) identified 56 records (Embase 19; PubMed 37). After deduplication (n=9), 47 records were screened; all were sought for retrieval, with 9 not retrieved (Embase 2; PubMed 7). Thirty-eight full-text reports were assessed; 11 were excluded (not DILI, n=9; multi-drug culprit unclear, n=1; insufficient detail, n=1). Twenty-seven case reports were included in the qualitative synthesis; with our case, the patient total is 28.


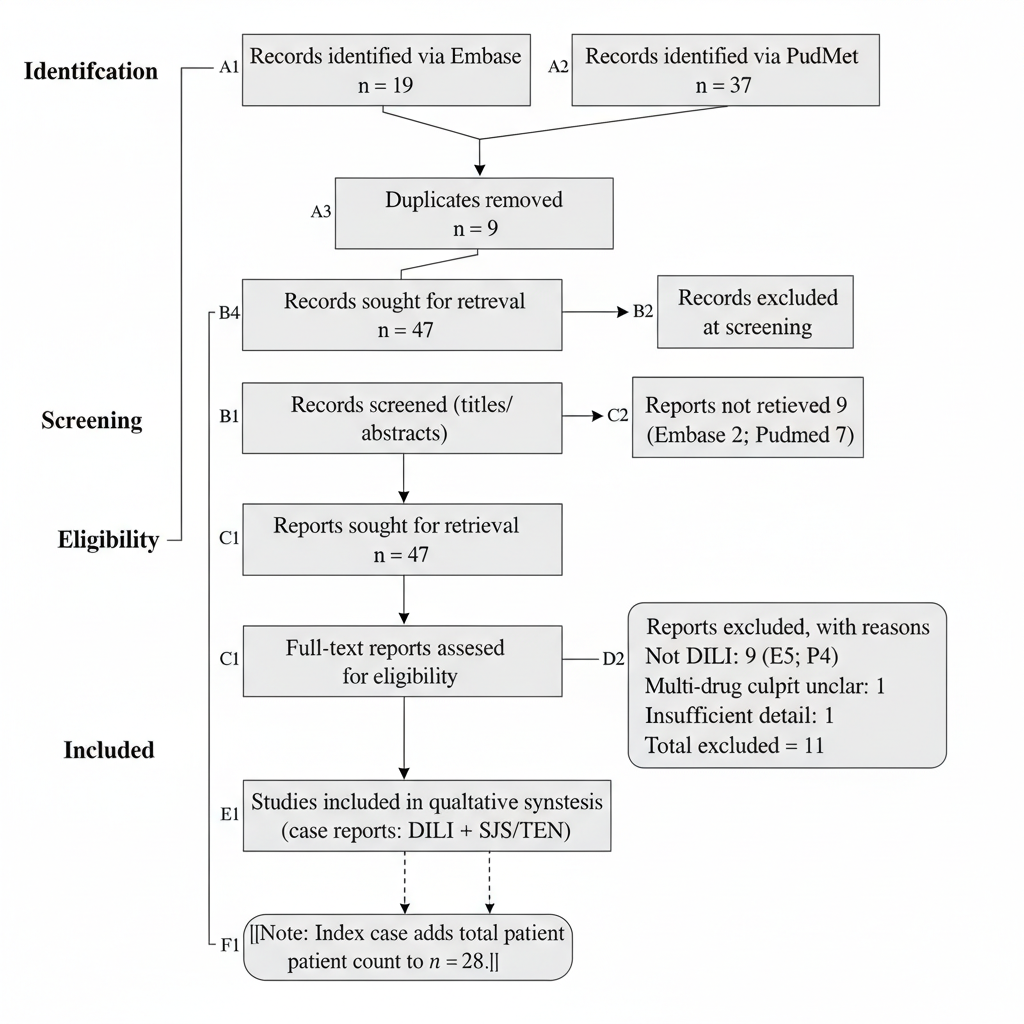

Supplement: Supplementary file 1 [file Table1.docx]
